# Effect of nurse-led intervention on postpartum wellbeing and parental competence among primi postnatal mothers at selected primary health centers, Chennai

**DOI:** 10.6026/973206300213654

**Published:** 2025-10-31

**Authors:** Jebamalar Rajan, Shankar Shanmugam Rajendran, Vanitha Narayanasamy Naidu, Gnana Sarjana Ramakrishnan, Anitha Parasuraman, Malathi Dhakshnamoorthy, Bibilin Wilson

**Affiliations:** 1 Department of Community Health Nursing, College of Nursing, Madras Medical College, The Tamil Nadu Dr. MGR Medical University, Chennai, Tamil Nadu, India

**Keywords:** Postpartum depression, parental competence, nurse-led intervention, primi-parous mothers, maternal well-being

## Abstract

The importance of structured support for primiparous mothers during the postpartum period is critical for improving their physical, emotional, and psychological well-being. Nurse-led interventions focusing on nutrition, newborn care and infection prevention significantly reduce postpartum depression and improve well-being and parental competence. The experimental group demonstrated notable improvements, including reduced depression scores and enhanced well-being and competence. Sociodemographic factors also played a role in influencing these outcomes, highlighting the need for tailored interventions. Thus, we show that nurse-led programs are an effective and scalable approach to improving maternal health and addressing postpartum challenges.

## Background:

The postpartum period, known as the "fourth trimester," is a critical stage for primi-parous mothers as they adjust physically, emotionally, and to new parenting roles [1]. Despite its importance, this period often receives insufficient attention, leading to various health and psychological challenges. Women recovering from vaginal delivery face perineal damage, while those with caesarean sections deal with prolonged surgical recovery [2]. Postpartum fatigue, affecting more than 60% of women in the first month, significantly impairs caregiving abilities. Hormonal fluctuations can also lead to symptoms such as nocturnal hyperhidrosis and alopecia. Complications like postpartum hemorrhage (1-5% of women) and infections, such as mastitis, further stress mothers [3]. The period is marked by mood changes, from temporary "baby blues" to more severe postpartum depression (PPD). "Baby blues" affect 49.6% of women and typically resolve within two weeks, while PPD affects 10-20% and requires psychiatric care. PPD hampers bonding and maternal confidence, especially for first-time mothers [4]. Emotional stress is aggravated by societal pressures and lack of experience. About 33.5% of primi-parous mothers face breastfeeding issues, including latching problems, nipple pain, and low milk supply. These challenges, coupled with fatigue and emotional strain, lead to feelings of inadequacy [5]. Relationship tensions may also arise, affecting mental health. Access to postpartum care plays a crucial role in maternal outcomes, but 30% of women globally lack essential care, particularly in low-resource areas. Postnatal services are vital for managing complications, supporting mental health, and educating about newborn care [6]. Without these services, mothers are at greater risk for complications and long-term mental health issues. Nurse-led interventions have shown significant benefits in improving outcomes. Structured educational programs increase maternal knowledge, confidence, and caregiving skills, reducing anxiety and depression [7]. These interventions boost maternal self-efficacy by 30-40%, promoting better parenting outcomes. Nutritional counseling helps mothers meet dietary needs, reducing fatigue. Education on neonatal care, such as breastfeeding, sleep, and infant cues, improves maternal confidence by up to 35% and reduces early breastfeeding cessation [8]. Given these benefits, it is important to assess the effectiveness of nurse-led interventions in improving postpartum well-being and parental competence among first-time mothers [9]. Therefore, it is of interest to evaluate the effectiveness of nurse-led interventions in enhancing postpartum well-being and improving parental competence among primi-parous mothers.

## Materials and Methods:

This quasi-experimental, non-randomized controlled study aimed to assess the efficacy of nurse-led interventions on postpartum well-being and parenting competence in primiparous mothers. The intervention group underwent structured nurse-led instruction, while the control group received conventional postpartum care. The research was conducted over a duration of four weeks at designated Primary Health Centres in Chennai, India.

The sample size was calculated using the formula:

N = (Z^2^xP(100-P))/L^2^

where Z = 1.96 (for 95% confidence), P = 12% (prevalence of postpartum depression), and L = 12% (absolute precision). This yielded a required sample size of 29 per group, which was rounded to 30, totaling 60 participants participants were selected through non-probability convenience sampling and provided informed written consent. Pre-test assessments were conducted using the Edinburgh Postnatal Depression Scale (EPDS), Short Form-36 Health Survey (SF-36), and the Parental Sense of Competence Scale (PSOC). These validated tools measured mental well-being, overall health, and parental competence, respectively. Data collection was performed at baseline (Day 1) and after the intervention (Day 21). The control group received routine postpartum education, while the experimental group received nurse-led interventions. The intervention included a 40-45-minute nurse-led educational session covering four components: nutrition (including iron and calcium-rich diets), infection prevention (e.g., hygiene, wound care), healthy practices (e.g., physical activity, postpartum contraception), and newborn care (e.g., breastfeeding, sleep patterns, and recognizing infant cues). Parental competence was supported through individualized assessment, psychological support, and guided practice. Sessions were structured and supported by printed educational materials. Data were entered in Microsoft Excel and analyzed using IBM SPSS Version 26. Descriptive statistics (mean, standard deviation, frequencies) were used for demographic and clinical data. Chi-square tests assessed group differences and associations, while paired t-tests compared pre- and post-intervention scores. Significance was set at p ≤ 0.05. Results were illustrated using bar diagrams and error plots to support statistical findings.

## Results:

The mean age of participants was 22.9 ± 2.4 years. All participants were female, married, and urban residents. Secondary education was the most common level (33.33% experimental, 30.00% control), and a majority were employed in government jobs. Monthly income was below ₹10,000 for 53.33% of the study group and 60.00% of the control group. Joint families were more common among experimental group mothers (56.67%), while nuclear families predominated in the control group (50.00%). Most followed a non-vegetarian diet and led sedentary lifestyles. Clinically, 80% of the study group and 86.67% of the control group had vaginal deliveries with episiotomy. Labor durations of 10-12 hours were reported by the majority, and analgesics were routinely used. Hospital stays of 3-5 days were typical. No participants experienced postpartum complications. A large proportion of mothers were functionally dependent at discharge. Before intervention, both groups had high depression levels (mean EPDS: 20.97 ± 1.2 in the experimental group and 21.13 ± 1.1 in the control group). Well-being scores (SF-36) were moderate (54.90 ± 4.6 and 55.27 ± 4.3), and parental competence (PSOC) scores were low (46.73 ± 3.8 and 47.20 ± 3.5) in both groups. Post-intervention assessments showed marked improvements in the experimental group. Depression scores decreased to 11.40 ± 1.5, well-being scores increased to 77.37 ± 5.1, and parental competence rose to 70.33 ± 4.2. In contrast, the control group showed only marginal changes across all domains. Paired t-tests showed significant pre-post differences within the experimental group (p = 0.001), but not in the control group. Between-group posttest comparisons also revealed statistically significant improvements in all measured outcomes favoring the effectiveness of nurse-led intervention on postpartum well-being and parental competence (Table 1 - see PDF, 2 - see PDF & 3 - see PDF). Significant associations were observed between Sociodemographic and clinical variables and posttest outcomes. Age, family income, occupation, type of family, and labor duration were significantly associated with postpartum depression, well-being, and competence (p < 0.05). Functional status was also linked to both well-being and depression scores. However, type of delivery, hospital stay, and postpartum complications were not significantly associated with any outcome variable.

## Discussion:

This study demonstrated that nurse-led interventions significantly enhance postpartum well-being and parental competence among primi-parous mothers. The intervention, which included structured education on nutrition, infection prevention, newborn care, and psychological support, led to substantial reductions in postpartum depression and improved self-reported health and parenting confidence. These findings are consistent with prior studies that highlight the value of structured educational support in improving maternal outcomes during the fourth trimester. In this study, the intervention group exhibited a significant reduction in mean depression scores, with a 31.90% improvement, and notable gains in well-being and parental competence. Shimpuku *et al.* [10] found similar effects where prenatal education decreased postpartum depression and increased maternal confidence in a Japanese cohort. Likewise, Khatun *et al.* [11] demonstrated improvements in fatigue, depressive mood, and maternal functioning among Bangladeshi mothers following a nurse-led postpartum care program. The high prevalence of depression observed in these study alligns global trends, where postpartum mood disorders affect 10-20% of new mothers [12]. A lack of support, physical exhaustion, and perceived inadequacy in parenting frequently intensify these psychological challenges. Du *et al.* [13] highlighted that reduced well-being strongly correlates with increased depression in primiparous women, further validating our findings. This study also explored associations between Sociodemographic and clinical factors with postpartum outcomes. Younger mothers (ages 20-25), those with joint family support, and those with higher functional independence reported better well-being and parental competence. These associations support the findings of Gashi *et al.* [14] who demonstrated that social support structures and early caregiving environments have a significant influence on parental self-efficacy. Additionally, Guo *et al.* [15] emphasized the importance of caregiver education in improving maternal confidence, particularly in managing newborn behaviors and breastfeeding, a finding echoed in this study. These results suggest that nurse-led interventions not only improve immediate postpartum outcomes but may also mitigate risks for long-term maternal mental health issues. Given the simplicity, cost-effectiveness, and scalability of this model, it should be considered an essential component of routine postpartum care. Further research could evaluate longitudinal outcomes and integration into digital health platforms to extend reach and accessibility.

## Conclusion:

Nurse-led interventions significantly improve postpartum well-being and parental competence among primi-parous mothers. Structured education on nutrition, hygiene, self-care, and newborn care effectively reduced depressive symptoms and enhanced maternal confidence. The data also highlighted significant associations between demographic factors such as age, income, and family structure and maternal outcomes. Therefore, it is of interest the integration of nurse-led programs into routine postnatal care as a cost-effective strategy to promote maternal mental health and parenting efficacy.
